# A natural chalcone induces apoptosis in lung cancer cells: 3D-QSAR, docking and an *in vivo*/*vitro* assay

**DOI:** 10.1038/s41598-017-11369-9

**Published:** 2017-09-06

**Authors:** Gang Chen, Di Zhou, Xue-Zheng Li, Zhe Jiang, Chengyu Tan, Xiu-Yan Wei, Junhong Ling, Jing Jing, Fen Liu, Ning Li

**Affiliations:** 1 School of Traditional Chinese Materia Medica, Shenyang Pharmaceutical University, Shenyang 110016, China; State Key Laboratory for Chemistry and Molecular Engineering of Medicinal Resources, Guangxi Normal University, Guilin, China; 2 Department of Pharmacy, Yanbian University Hospital, Yanji, 133000 China; 3 College of Marine Technology and Environment, Dalian Ocean University, Dalian, China; 4 Department of Pharmacology, Shenyang Pharmaceutical University, Shenyang, 110016 China; 5 School of Pharmaceutical Engineering, Shenyang Pharmaceutical University, Shenyang, 110016 China

## Abstract

This study was to study the antitumor effect of lonchocarpin (**34**) from traditional herbal medicine *Pongamia pinnata* (L.) Pierre and to reveal the underlying mechanism. The cytotoxic activities of lonchocarpin were evaluated in 10 lung cancer cell lines and it exhibited 97.5% activity at a dose of 100 μM in the H292 cell line. A field-based quantitative structure-activity relationship (3D-QSAR) study of 37 flavonoids from *P*. pinnata was also performed, and the results obtained showed that the hydrophobic interaction could be the crucial factor for the antitumor activity of lonchocarpin. Molecular docking studies revealed that lonchocarpin bound stably to the BH3-binding groove of the Bcl-2 protein with hydrophobic interactions with ALA146. Also, lonchocarpin significantly reduced cell proliferation via modulating Bax/Caspase-9/Caspase-3 pathway. An apoptotic test using flow cytometry showed that lonchocarpin produced about 41.1% and 47.9% apoptosis after treatment for 24 h and 48 h, respectively. Moreover, lonchocarpin inhibited tumor growth in S180-bearing mice with an inhibition rate of 57.94, 63.40 and 72.51%, respectively at a dose of 25, 50 and 100 mg/kg. These results suggest that lonchocarpin is a potentially useful natural agent for cancer treatment.

## Introduction

Flavonoids are one of the most extensively investigated natural products since they are distributed widely in nature. Flavonoids have been reported to possess potent cytotoxic activity against a number of cancer cell lines^1–3^. Among flavonoids, chalcones, either natural or synthetic, have been reported to possess *in vitro* antitumor activities^4–8^. However, the plausible mechanism and the *in vivo* antitumor effect of a certain natural chalcone has largely remained unknown. Thus, this study focused on one natural chalcone, lonchocarpin, with high cytotoxic activity in 10 human lung cancer cell lines, from the root of *Pongamia pinnata* (L.) Pierre. To fully understand its antitumor properties, field-based 3D-QSAR, docking, flow cytometry, apoptosis-related gene expression in the H292 human lung cancer cell line and *in vivo* antitumor experiments involving lonchocarpin were performed.

Lonchocarpin exhibits cytotoxic effects against the CEM leukaemic cell line (IC_50_ value 10.4 μg/ml)^9^ but its cytotoxic effects in other tumor cells, especially lung cancer cells, have not been reported. Recent research involving synthetic chalcones proved that they could induce apoptosis in A549 human lung cancer cells^10^. However, to the best of our knowledge, there have been no detailed investigations of key substituents or the mechanism for the cytotoxic activity of natural flavonoids in H292 cells.

In present study, the growth inhibitory activitis of lonchocarpin were evaluated in ten different human lung cancer cell lines for the first time. Lonchocarpin was more cytotoxic to H292 cells than A549 cells and the flow cytometry results suggested that lonchocarpin induced apoptosis in H292 cells. Western blot analysis confirmed the involvement of Bax and Bcl-2 protein alterations in lonchocarpin-induced apoptosis in H292 cells. The hallmarks of cancer consist of six biological effects, including ever-lasting signals for proliferation, escaping from growth suppressors, opposing cell death, being replicative immortality, induction of angiogenesis and activation of invasion and metastasis^11^. In the case of oncogenesis, tumor cells oppose apoptotic signals so as to avoid cell death. The counterbalance of proapoptotic and antiapoptotic regulatory proteins of Bcl-2 family plays a “crucially important role” in cell apoptosis. Defined as inhibitors of apoptosis, Bcl-2 along with Bcl-xL, Bcl-w, Mcl-1 and A1 that are the closest relatives of Bcl-2, bind to two proapoptotic triggering proteins, Bax and Bak, in the first place so that the Bak and Bax are restrained. Once relieved of the restrain, Bax and Bak compromise the integrity of the outer mitochondrial membrane, and thereby release proapoptotic signaling factors, such as cytochrome c that can trigger the activation downstream genes like caspase-9 and caspase-3^12^. Thus, targeting Bcl-2 via binding to its BH3-binding groove provides a possible strategy for inducing tumor cell apoptosis and several Bcl-2-inhibitory anticancer agents have been reported^13, 14^. Therefore, in this study, the Bcl-2 binding potency of lonchocarpin and other active flavonoids from *P*. *pinnata* (L.) Pierre were also studied in a docking experiment with Glide since both the Bax up-regulation and caspase-3 activation are involved in lonchocarpin-induced apoptosis.

## Results

### Cytotoxic properties of the isolated compound

**Figure 1:**
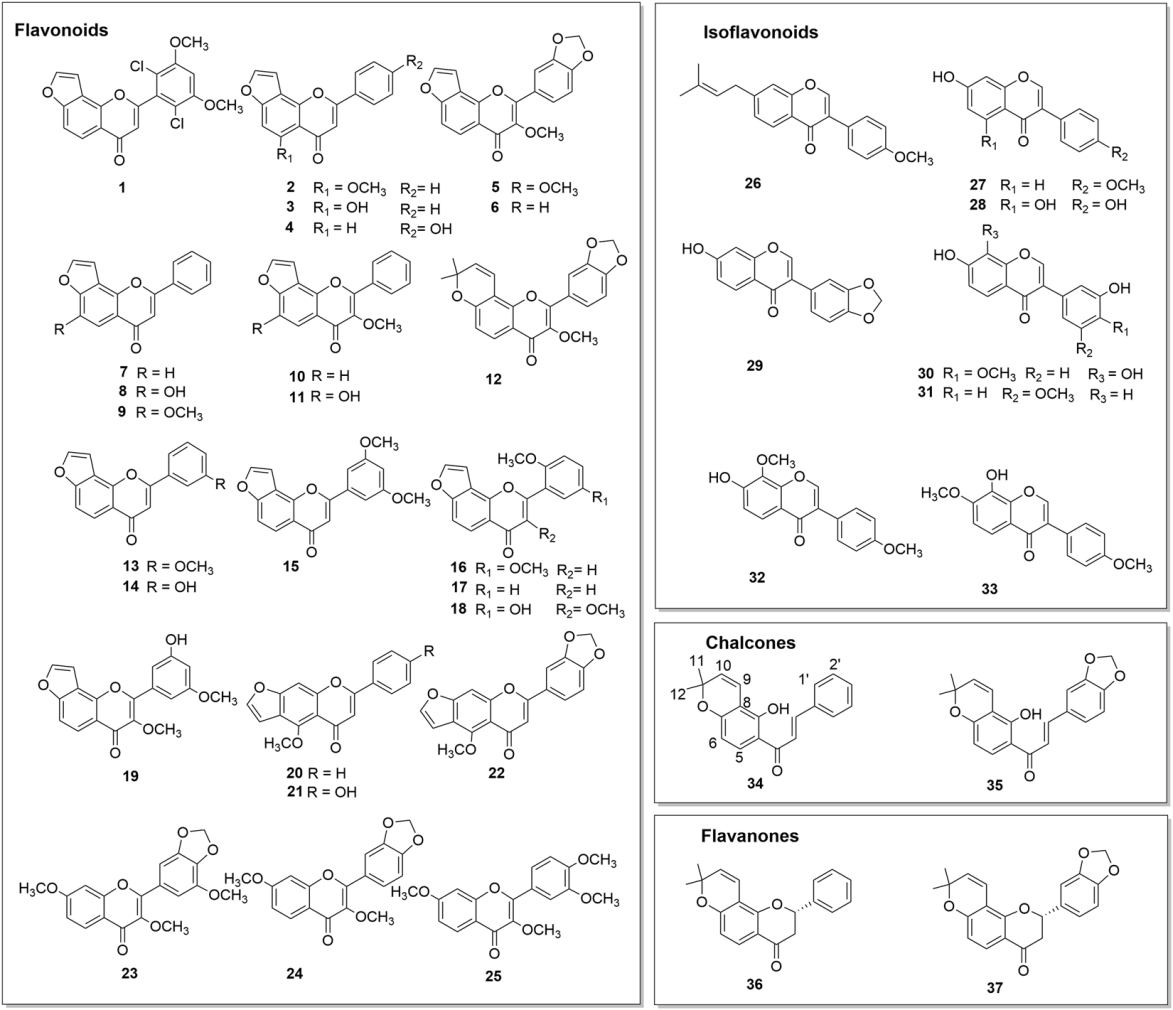
Structures identified from *Pongamia pinnata* (L.) Pierre.

Thirty-seven flavonoids were isolated from the root of *Pongamia pinnata* (L.) Pierre (Fig. 1). The cytotoxic activity of lonchocarpin (**34)** against ten human lung cancer cell lines was evaluated using 3-(4,5-dimethylthiazol-2-yl)−2,5-diphenyl tetrazolium bromide (MTT) method. Lonchocarpin showed potent inhibition of H292, H522, SW1573, H460, H1944, and H226 cell lines and moderate cytotoxic activity in H358, A549, H1792, and Calu-1 cell lines as shown in Supplementary Table S1. The compound showed 97.5% inhibitory activity at 100 μM (IC_50_ value 10 μM) in H292 cells but no cytotoxicity at 1000 μM in Vero cells. Thus, we chose the H292 cells for further antitumor evaluations of the other 36 flavonoids from *Pongamia pinnata* (L.). All the IC_50_ values of the 37 flavonoids can be found in Supplementary Table S2. Compounds **35**-**37** showed moderate cytotoxic activity with IC_50_ values of 61.5, 50.0, 61.5 μM and compounds **12**, **15**, **23**, **26**, **33** were only weakly cytotoxic with IC_50_ values of 100.0, 158.5, 311.9, 293.8 and 215.8 μM. The remaining compounds had IC_50_ values of over 300 μM.

### Gaussian-based 3D-QSAR analysis

**Figure 2:**
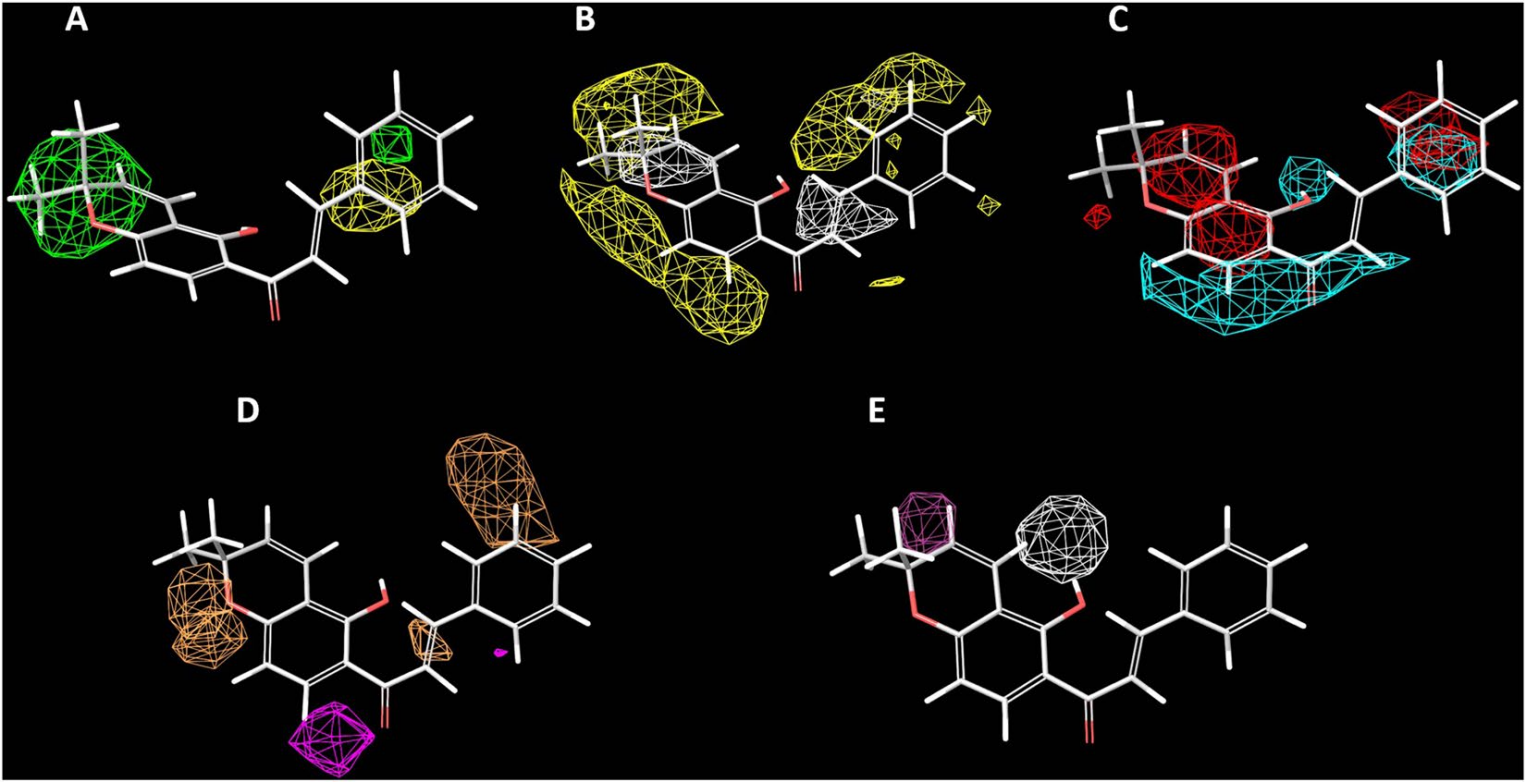
Contour maps obtained for the Gaussian-based 3D-QSAR model based on lonchocarpin. (**A**) For Gaussian steric, green contours are areas where the steric interaction is favored and yellow contours represent the region where steric interaction is disfavored. (**B**) For gaussian hydrophobic, yellow and white contours represents favored and disfavored hydrophobic interacting regions. (**C**) For gaussian electrostatic, cyan and red contours represent the favorable electropositive and favorable electronegative regions. (**D**) For HBA, orange and magenta contours represent the favorable and unfavorable hydrogen bond acceptor interactions. E For HBD, white and maroon contours represent favorable and unfavorable hydrogen bond donor interactions.

The field-based QSAR tool of Schrodinger 2013 was employed when constructing 3D-QSAR models that can help understand the relationship between the chemical groups in a three-dimensional grid and the cytotoxic activity. Using PLS and taking five structurally important features into account, the Gaussian-based QSAR model was established on the basis of correlating with the five features (steric, electrostatic, hydrophobic, HBD and HBA) of the training set. The predicted pIC_50_ values are given in Supplementary Table S2 and Figure S1, and the contribution of five features represented as field intensities is given in Supplementary Table S3. The contributions of the steric, hydrophobic, HBD, HBA and electrostatic were 0.51, 0.23, 0.14, 0.07 and 0.05, respectively (see Supplementary Table S3). The steric and hydrophobic features gave much higher field intensities of 0.51 and 0.23, respectively, indicating that steric and hydrophobic features were essential for protein-ligand interactions. Consequently, we focused on the steric and hydrophobic properties in this QSAR model. The contour maps given by the Gaussian-based 3D-QSAR model based on lonchocarpin are given in Fig. 2. Figure 2A are the contour map which represents steric interactions in green and yellow colors. Methyl substitutions in ring D of **12**, and **34**–**37** showed higher activity than others and two methyl substitutions of ring D were located in the favorable green region as shown in Fig. 2A. This indicated the important role of carbon stereochemistry in the activity, explaining why almost all the natural benzopyran flavonoids (**12**, **34**–**37**) had a higher activity than natural benzofuran flavonoids. Figure 2B shows the hydrophobic contours. The sp^2^ protons in ring A, C and D were in yellow color region where hydrophobic groups may increase the activity, which means that active flavonoids (**12**, **34**–**37**) were expected to bind into an active pocket where strong hydrophobic interactions between the target protein and active flavonoids could take place.

### Analysis of apoptosis by flow cytometry

**Figure 3:**
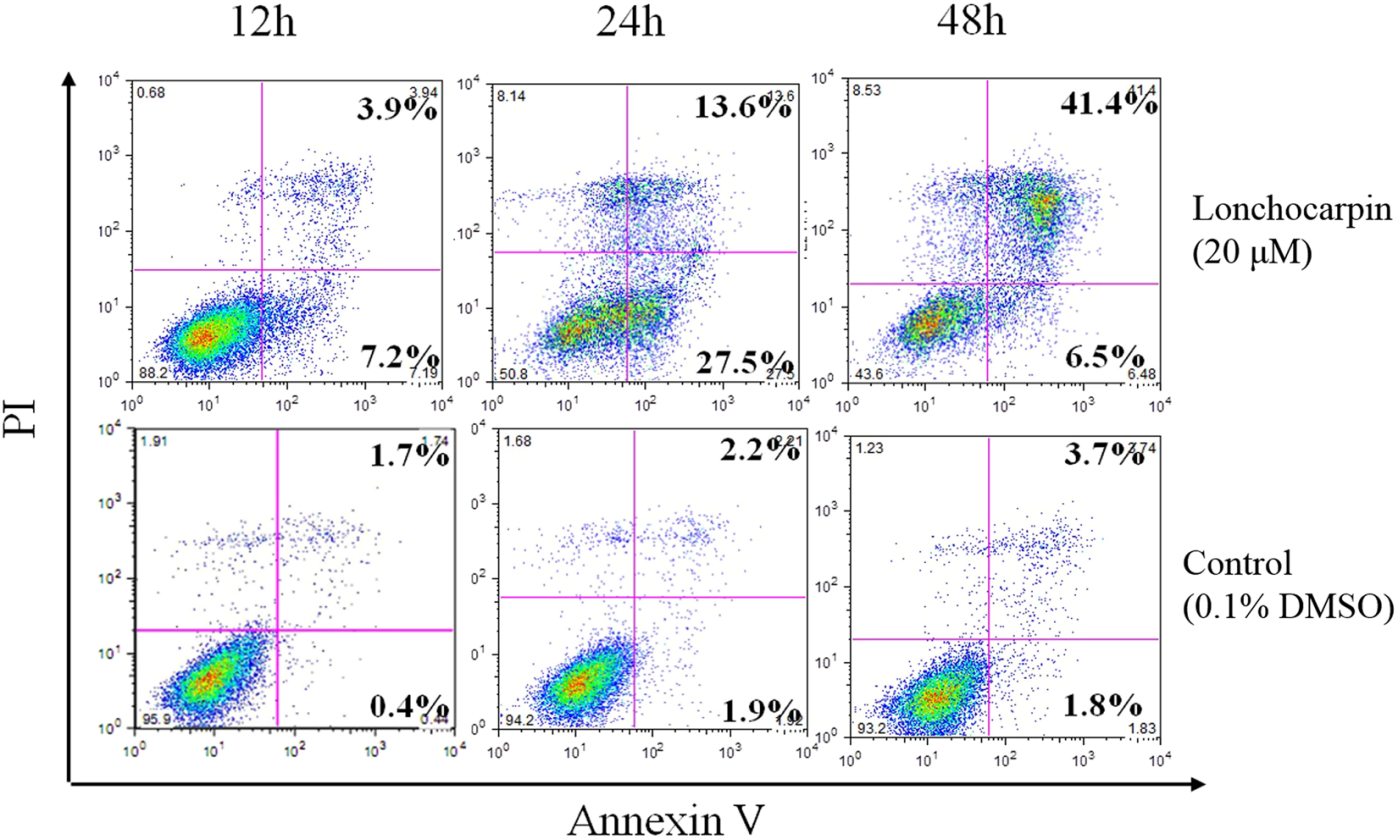
Lonchocarpin induces apoptosis in H292 cells. Flow cytometric analysis was used to determine the cell apoptosis in H292 cells after various times (12 h, 24 h, 48 h) with 20 μM lonchocarpin by Annexin V-FITC and PI staining.

Lonchocarpin significantly inhibited H292 proliferation as reported above. However, the mechanism whereby H292 cells were inhibited remained unknown. Thus, cell apoptosis was examined by annexin V/PI staining assay. After a 12 h treatment, lonchocarpin at a dose of 20 μM caused 7.2% early apoptosis and 3.9% late apoptosis in H292 cells (Fig. 3). Compared with the control group, lonchocarpin produced about 41.1% and 47.9% apoptosis after administration for 24 h and 48 h, respectively. Also, early apoptotic cells were mainly found following treatment with lonchocarpin for 12 h and 24 h. Thus, inducing apoptosis of H292 cells was considered to be the underlying mechanism of the cytotoxic effects of lonchocarpin.

### Effect of lonchocarpin on caspase-3 activity in H292 cells

**Figure 4:**
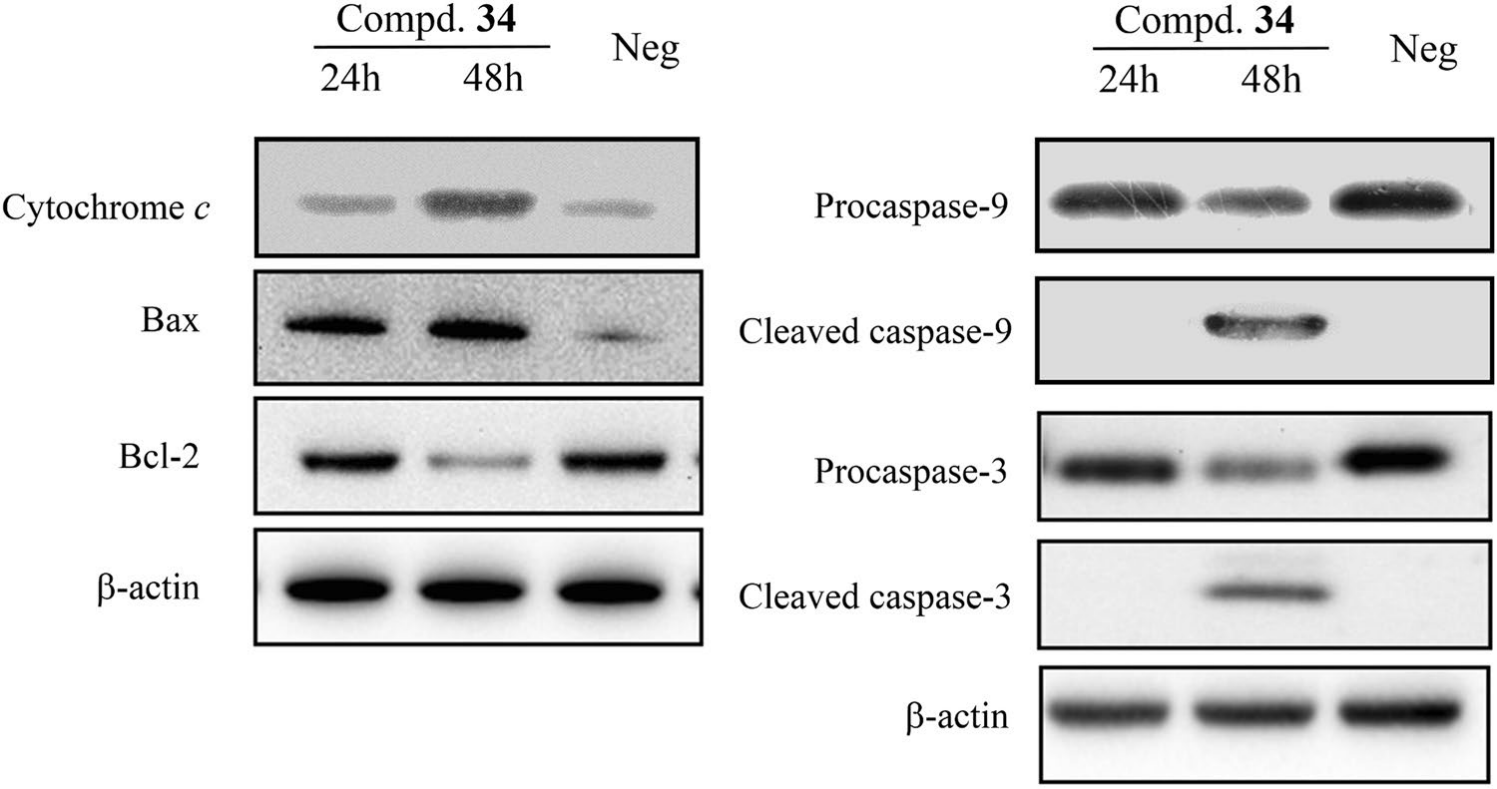
Lonchocarpin modulates caspase-3 activation via Bax/caspase-9 pathway. Representative immunoblots of Cleaved caspase 3 and caspase 9 (right), Pro-caspase 3 and caspase-9 (right), cytochrome *c* (left), Bax (left) and Bcl-2 (left) in H292 cells; Human lung cancer cells H292 were pretreated with DMSO (as a negative control) and lonchocarpin at 20 μM for 24 h and 48 h. Neg: negative control.

Programmed cell death is precisely regulated by many factors, including the synergetic modulation of pro-survival and pro-apoptotic genes. When apoptosis is triggered in cells, permeability of mitochondrial membrane is increased and cytochrome *c* that cause the subsequent chain reaction of destructive protease in the cytoplasm is released. Featured either by their function in determining cell fate, or by their structural characters of the Bcl2 homology (BH) motifs, Bcl2 family members are the key regulators controlling the permeability of mitochondrial outer membrane. Among the Bcl-2 protein family, Noxa, Bad, Bax, Bak and Puma are proapoptotic while Bcl-2 and Bcl-xL are antiapoptotic. Bax and Bak that have four different BH motifs homo-oligomerize when activated to form pores in the mitochondrial outer membrane, initiating apoptosis in cells. Though structurally resembles those of proapoptotic proteins, pro-survival homologs, such as Bcl-xL, Bcl-2, Bcl-w, Mcl-1, Bfl-B and Bcl-1 in humans, resist apoptosis through binding to and thereby restraining Bax and Bak. Through cleavages, caspases could be activated sequentially from inactive forms and caspase-3, one of the key activated protease during the initial phase of apoptosis, activates other caspases as well as other related proteins in either nucleus or cytoplasm proteolytically. To investigate the mechanism underlying lonchocarpin-induced apoptosis in H292 cells, we focused on the pathway of the Bcl-2/Bax-mediated activation of caspase-3. Following incubation with 20 μM lonchocarpin for 24 h and 48 h, protein expression of Bcl-2, Bax, procaspase-3 and cleaved caspase-3 were detected by western blotting analysis. Lonchocarpin significantly increased cleaved caspase-3 at 48 h. The increased cleaved caspase-3 was associated with a reduction in procaspase-3. However, Bcl-2 protein was obviously down-regulated by lonchocarpin at 20 μM for 24 h and 48 h. And the reduction in Bcl-2 was associated with the up-regulation of Bax (Fig. 4), suggesting the activation of caspase-3 was Bax-mediated. Supporting this notion, treatment with 20 μM lonchocarpin also led to the up-regulation of cytochrome *c* and activation of caspase-9 (Fig. 4).

### Prediction of the common binding mode of identified active flavonoids by silico docking

**Figure 5:**
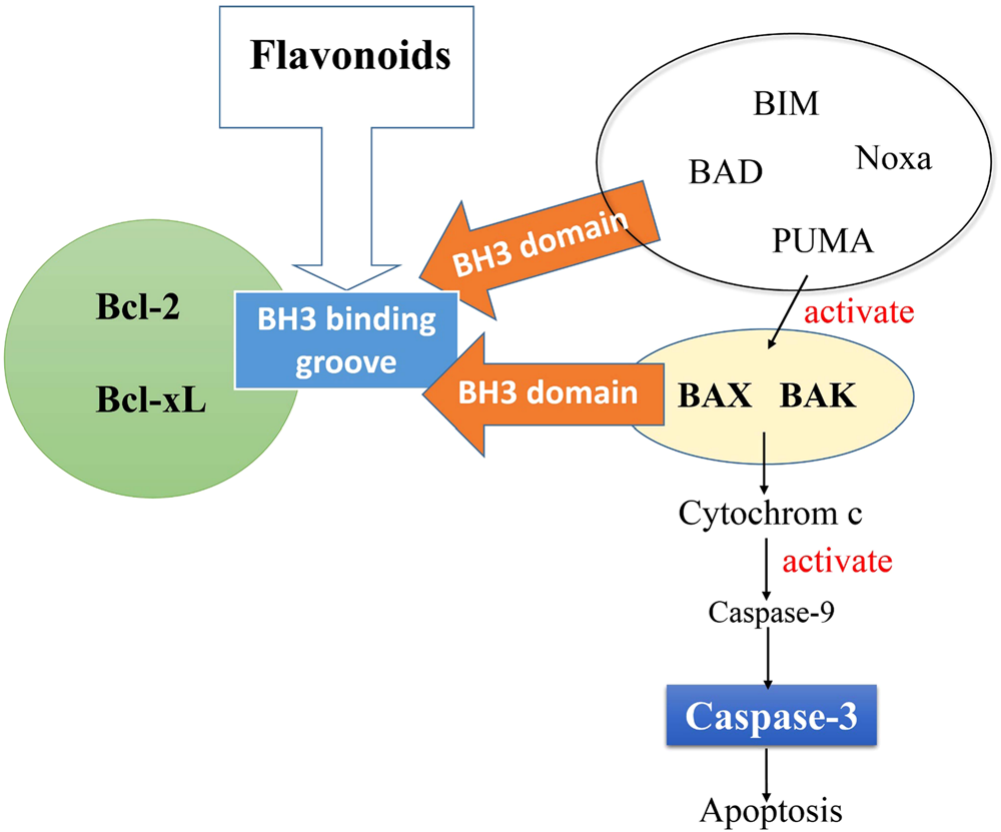
A predicted model for active flavonoid-induced apoptosis in H292 cells. Flavonoid induces cell apoptosis via inhibiting Bcl-2 and activation of caspase-3 dependant pathway. Lonchocarpin can occupy the BH3 binding groove, keeping them from restraining Bax and Bak. Bak or Bax can permeabilize the mitochondrial outer membrane and release cytochrome c, which results in activating caspase-9 and caspase-3.

So far up to four conserved amino acids stretches are found in Bcl-2 family proteins, known as BH (Bcl-2 homology) domains^15, 16^. There are four BH3-only proteins (Bad, Bim, Noxa and Puma) that capture signals of death and subsequently stimulate the downstream proapoptotic effectors Bak and Bax^17–19^. Bak and Bax that are in the active form can compromise mitochondrial and thereby release proapoptogenic effectors, such as cytochrome *c* that triggers the cleavage of caspase-9 and ultimately activates caspase-3^20^. The aforementioned processes are decided by the binding of proapoptotic effectors to the antiapoptotic ones via the BH3 domain/BH3-binding groove interaction^21^. For example, the BH3-only protein BAD disrupts the interaction of Bcl- xL or Bcl-2 with Beclin1 by binding to the BH3-binding groove^22^. Therefore, occupying the BH3-binding groove of the antiapoptotic members, such as Bcl-xL or Bcl-2, would definitely block the signal pathway, contributing to caspase-3 activation and triggering apoptosis (Fig. 5).

**Figure 6:**
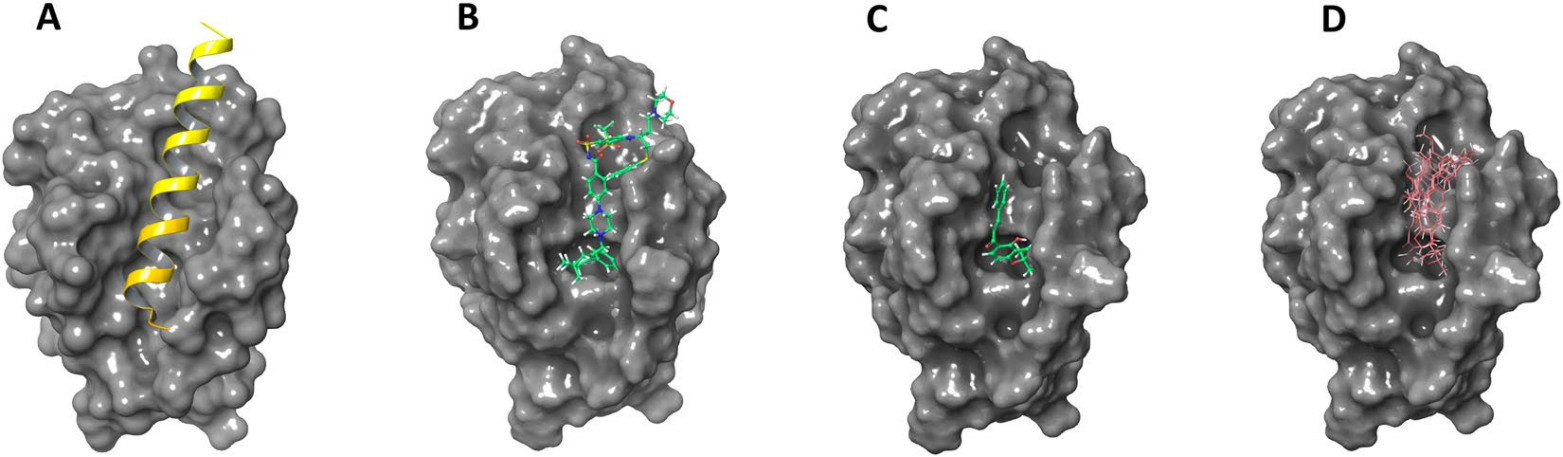
Predicted molecular interactions between the active flavonoids and the BH3-binding groove of Bcl-2. Bcl proteins are presented as gray cartoons of solid surfaces. (**A**) Crystal structure of Bcl-2 in complex with a Bax BH3 peptide represented by a yellow ribbon (PDB: 2XA0). (**B**) The binding site can be occupied by the synthetic chemicals as shown via the co-crystal structure of Bcl-2 and navitoclax (PDB: 4LVT). (**C**) Lonchocarpin is propoesd to occupy the same binding site. (**D**) Lonchocarpin and compounds **12**, **15**, **23**, **26**, **33** in pink color block this hydrophobic BH3-binding groove by a similar way.

**Figure 7:**
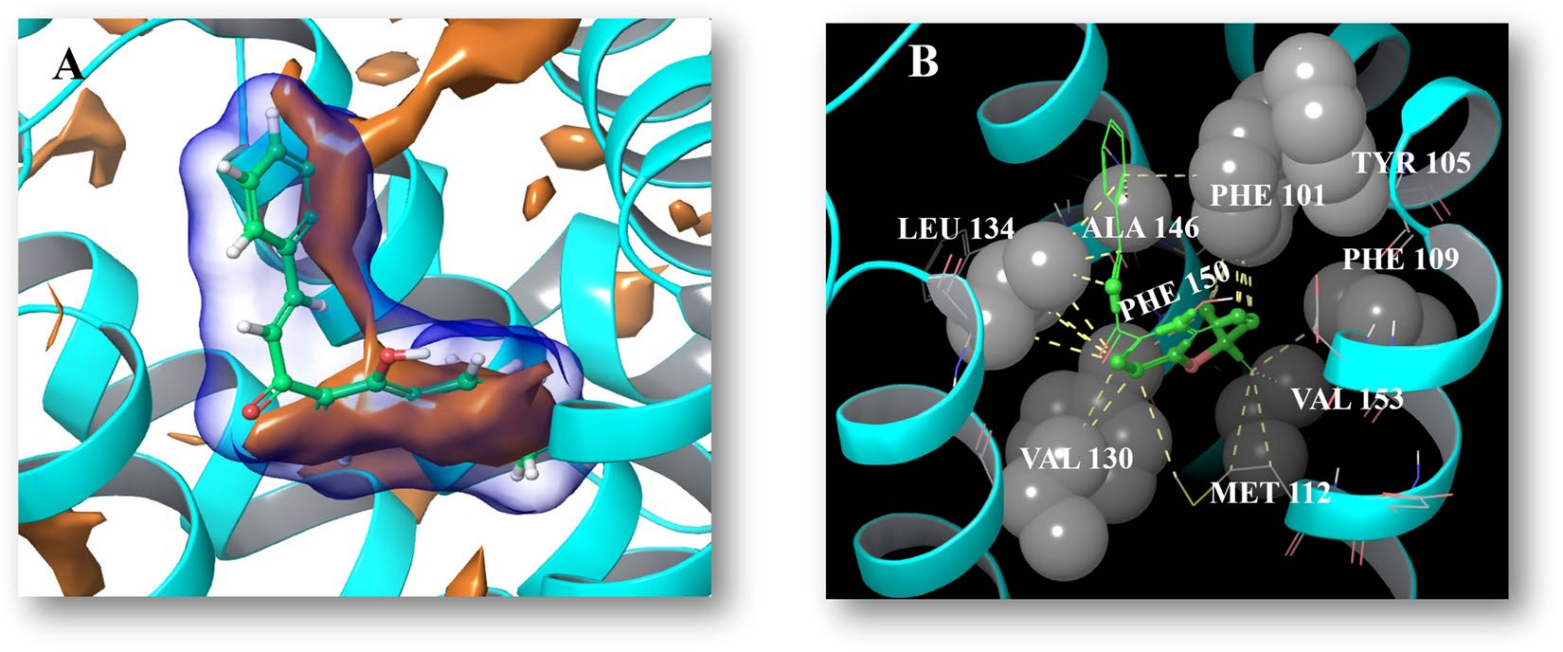
Predicted hydrophobic interactions between lonchocarpin and the BH3-binding groove of Bcl-2. (**A**) Hydrophobic surface represented in dark yellow color of lonchocarpin and Bcl-2 complex. (**B**) Hydrophobic interactions of lonchocarpin and Bcl-2 complex. The hydrophobic enclosure of Bcl-2 binding groove is represented by solid gray balls and the potential hydrophobic interaction of the ligand-protein complex is given by shot dashed yellow lines.

In order to clarify the potential mechanism by which flavonoids activate caspase-3 in H292 cells, a docking study of active flavonoids on the BH3-binding groove of the Bcl-2 protein was performed. The active flavonoids (**12**, **34**–**37**) were made to dock to the co-crystal structure of Bcl-2 with the ligand navitoclax, which is a selective inhibitor of Bcl-2 and Bcl-xL (PDB: 4LVT)^23^, using Glide. The C_6_-C_3_-C_6_ scaffold of the flavonoids has been consistently predicted to occupy the Bcl-2 hydrophobic binding groove and the specific binding region was overlapped with those of the synthetic navitoclax (Fig. 6B–D). As a result, complex formation with Bax (Fig. 6A) and thus signaling would be inhibited. The detailed hydrophobic interaction between lonchocarpin (**34**) which was the most active and the binding site was explored as shown in Fig. 7. Lonchocarpin is predicted to form hydrophobic interactions with the hydrophobic enclosure of Phe-101, Phe-109, Met-112, Val-130, Leu-134, Ala-146, Phe-150 and Val-153 (Fig. 7B). In addition, ring B of lonchocarpin exhibited a Pi-Pi interaction with Arg-143. An MM-GBSA calculation was employed to further evaluate the potential binding affinity of lonchocarpin. Navitoclax was redocked into the Bcl-2 binding pocket and the △G_Bind_ values of lonchocarpin and navitoclax were calculated by the MM-GBSA method. Lonchocarpin gave a △G_Bind_ value of −41.36 kcal, which showed a potentially moderate binding affinity compared with that of navitoclax which was −80.12 kcal. Additionally, more Bcl-2 protein structures (PDB: 1ysw, 2o2f, 2o21, 4aq3, 4ieh) were used to see if this binding mode was universal in terms of other Bcl-2 protein structures. The docking results proved that lonchocarpin inhibited the BH3-binding groove of the six Bcl-2 protein structures in a similar way and the stereo-view of the superimposed Bcl-2 protein structures is given in Supplementary Figure S2.

### *In vivo* antitumor activity of lonchocarpin in S180 implanted mice

Significant tumor inhibitory effect was observed in the lonchocarpin-treated groups at a dosage of 25, 50 and 100 mg/kg, with an inhibition rate at 57.94, 63.40 and 72.51%, respectively (Table 1). And, the anti-tumor activity of lonchocarpin showed a clear dose-effect relationship. Interestingly, the spleen index of the CTX group was greater than that of the control group. However, it has also been reported that CTX can cause splenomegaly in mice and subsequently increase the spleen index^24^. Therefore, a change in the spleen index might depend on the dosage and time of CTX administration.

**Table 1:** Effects of lonchocarpin on spleen index and tumor growth in S180-bearing mice (n = 8, mean ± SE) ***P < 0.001, compared to model groups.

Group	Dose[mg/kg]	Mouse number	Body weight/g		Tumor weight/g	Inhibitory rate (%)	Spleen index (mg/g)
			After experiment	Before experiment			
Model	0	8	27.43 ± 0.30	37.22 ± 0.48	8.05 ± 1.15	0	3.72
Lonchocarpin	25	8	26.45 ± 0.68	39.19 ± 2.15	3.39 ± 0.69^***^	57.94	3.08
	50	8	28.99 ± 0.47	36.48 ± 1.38	2.95 ± 0.66^***^	63.40	3.17
	100	8	28.21 ± 1.98	38.71 ± 2.20	2.21 ± 0.72^***^	72.51	3.30
CTX	25	8	28.09 ± 0.56	35.97 ± 0.89	1.55 ± 0.30^***^	80.75	4.77

## Discussion

As structurally revealed, the BH1–3 domains of antiapoptotic proteins such as Bcl-2 fold and form a globular domain that possesses a hydrophobic groove on the surface, where a pro-death BH3 domain of an amphipathic alpha helix of B24 residues can bind to and so as to prevent Bak or Bax from interrupting the intracellular membranes integrity^25, 26^. Even though flavonoids have been reported to induce apoptosis in cancer cells^27^, the chemico-biological interactions between the ligand and Bcl-2 hydrophobic groove remain obscure.

In this study, the field-based 3D-QSAR results suggested the hydrophobic interaction at C-4, C-5, C-11, C-12, C-1′ and the C-2′ region of lonchocarpin (**34**) (Fig. 1) would help improving the cytotoxic activity, which contributed 23% of the model (Fig. 2B). The docking study of lonchocarpin gave almost exactly the same result as the field-based 3D-QSAR hydrophobic model by showing a hydrophobic surface in dark yellow in the C-4, C-5, C-11, C-12, C-1′ and C-2′ region of lonchocarpin in the Bcl-2 docking complex at (Fig. 7A). The detailed hydrophobic interactions between C-4, C-5, C-11, C-12, C-1′ and the C-2′ region of lonchocarpin and the hydrophobic groove of Bcl-2 were given in Fig. 7B. As show in Fig. 6A the hydrophobic groove of Bcl-2 protein can interact and form a complex with a Bax BH3 peptide, which could be disrupted by a synthetic compound navitoclax and lonchocarpin in a similar way as shown in Fig. 6B, indicating that the hydrophobic groove of the pro-survival family member Bcl-2 may be a target for lonchocarpin-induced apoptosis in H292 cells and, thereby, Caspase-3 activation.

The △G_Bind_ value from the mm-gbsa calculation of lonchocarpin and the Bcl-2 complex was −41.36 kcal, which is higher than that of navitoclax, −80.12 kcal. However, lonchocarpin are of low toxicity to normal cells as it shows no toxicity up to 1000 μM in Vero cells. The *in vivo* antitumor activity of lonchocarpin in S180 implanted mice showed an inhibition rate of 57.94, 63.40 and 72.51% at a dosage of 25, 50, 100 mg/kg, confirming the potent *in vivo* tumor inhibitory potential of lonchocarpin at a higher dosage.

*Pongamia pinnata* (L.) Pierre, is a traditional herb, native to India and widely distributed in tropical Asia, Australia, Polynesia and the Philippines. Its diverse traditional uses have prompted scientists to investigate its pharmacological properties for therapeutic use. However, this is the first time a study has been carried out to examine the antitumor biochemical profile of *P*. *pinnata* and the underlying mechanism of action of its main bioactive constituent lonchocarpin.

In conclusion, lonchocarpin significantly inhibits tumor growth both *in vitro* and *in vivo* and its underlying mechanism of action involves Bcl-2 inhibiting induced Caspase-3 activation. The hydrophobic groove of Bcl-2 protein is considered to be the target for lonchocarpin preventing Bcl-2 from binding the pro-death BH3 domain of Bax or Bak.

## Materials and Methods

### General experimental procedures

NMR data were collected in Bruker ARX-400 and ARX-600 spectrometers, and TMS was used as the internal standard. HR-ESI-MS spectrum was recorded in m/z (rel. %) mode by Bruker micro TOF-Q mass spectrometer. Silica gel used in the experiment was purchased from Qingdao Ocean Chemical Group Co. of China. HPLC separations were carried out on Shimadzu HPLC apparatus (Shimadzu RID-20A UV detector and Shimadzu LC-6AD series pumping system) with a YMC-pack ODS column (250 × 20 mm). Primary antibodies against Bcl-2, Bax, caspase 3 and cleaved caspase-3; Dimethyl sulfoxide (DMSO), thiazolyl blue (MTT), CDCl3, DMSO-*d*_6_ and Pyridine-*d*_5_ were afforded by Sigma-Aldrich Company (St. Louis, MO, USA). Cyclophosphamide (CTX) was ordered from HengRui Medicinal Limited Compony (Jiangsu, China). Sodium chloride injection (Batch No. 100232) was bought from Collen Cornell pharmaceutical compony (Jilin, China). DDP was purchase from Sigma-Aldrich Company (St. Louis, MO, USA). RPMI-1640 medium was purchased from GIBCO (NY, U.S.A.)

### Plant material

The stem and branch of *P*. *pinnata* were afforded by Professor Chunyan Yan of (Guangdong Pharmaceutical University) in this experiment. The voucher specimen (No. 20100628) is deposited in School of Traditional Chinese Meterial Medica, Shenyang Pharmaceutical University.

### Extraction, isolation and identification of the compounds 1–37

The tested compounds were purified from Dry stems of *P*. *pinnata* (L.) Pierre. The methods of extraction, isolation and identification has been described in Supplementary Data. The purity of the identified compounds **1–37** were determined by HPLC analysis before the bioassay described in this paper, Compounds **1** (98.9%), **2** (99.1%), **3** (98.2%), **4** (98.9%), **5** (98.8%), **6** (99.0%), **7** (98.7%), **8** (98.8%), **9** (98.7%), **10** (99.3%), **11** (98.1%), **12** (98.7%), **13** (98.6%), **14** (98.5%), **15** (98.7%), **16** (98.2%), **17** (98.9%), **18** (99.1%), **19** (98.4%), **20** (98.2%), **21** (98.7%), **22** (99.1%), **23** (98.9%), **24** (99.2%), **25** (98.7%), **26** (99.3%), **27** (98.2%), **28** (98.5%), **29** (98.7%), **30** (98.8%), **31** (98.3%), **32** (99.1%), **33** (98.7%), **34** (98.6%), **35** (98.6%), **36** (98.7%), **37** (98.1%).

### Animals and cell lines

Male Kunming mice weighing 18–22 g, were afforded by the Animal Experiment Center of Shenyang Pharmaceutical University (SCXK2015–0001). The mice were housed according to the standard principles of the animal ethical committee of Shenyang Pharmaceutical University in the Animal Experiment Center. Murine sarcoma S180 cells were supplied by Porf. Xiuyan Wei (Shenyang Pharmaceutical University) and reproduced in our lab.

A549, H292, H460 and H1792 cell lines were donated by Professor Ma Xiaochi (Dalian Medicinal University) and cultured in our laboratory. The cells were cultured by RPMI-1640 medium, which was supplemented with fetal calf serum (10%), penicillin (100 IU/mL), streptomycin (100 mg/L) and L-glutamine (0.03%). The cells were maintained at 37 °C with 5% CO_2_ in a cell incubator.

H226 (TCHu235), H358 (TCHu151), and Calu-1 (TCHu192) cell lines were afforded by the Cell Bank of the Chinese Academy of Sciences (Shanghai, China). H1944 (CBP60066), H522 (CBP60140), and SW1573 (CBP60177) cell lines were afforded by Cobioer Biosciences Co., LTD (Nanjing, China).

### Cytotoxicity bioassay

The cytotoxicities of the tested flavonoids were assayed in ten human lung cancer cell lines (A549, H226, H358, H522, H292, H1944, H460, H1792, SW1573, and Calu-1) using MTT reduction assay^28^. Cisplatin (DDP) was selected as a positive control. Compounds **1**–**37** were dissolved in DMSO to obtain a clear solution of 1 M, which was diluted to 400 mM, 200 mM, 100 mM, 75 mM, 50 mM, 37.5 mM, 25 mM, 10 mM and 1 mM respectively as stock solutions. The DMSO concentration in the medium was kept below 0.10%. Moreover, the stability of tested compounds in experimental buffer was confirmed at 24 h and 48 h respectively by means of HPLC analysis. Then the human lung cancer cells were placed in 96-well culture plates (5 × 10^4^ cells/well) and incubated for 24 h. Then, the cells were pretreated with different concentrations of test samples (gradient concentrations 1.0, 10.0, 25.0, 37.5, 50, 75, 100, 200, 400, 1000 μM for compounds **1**–37 and DDP) for 48 h. And cell growth was measured by MTT assays. The cytotoxicities against the lung cancer cells were determined systematically and expressed as IC_50_ value.

### Field based 3D QSAR (FQSAR)

The field-based method resembles CoMFA^29^ and CoMSIA^30^ with some modifications. First, OPLS_2005^31, 32^ force field was used to generate electrostatic and steric fields while CoMFA use Tripos force. Second, a 30 kcal/mol threshold was used for both the van-der-Waals and electrostatic interactions. Third, data were scaled according to the maximum potential divided by the standard deviations of the field over whole training set. Phase H-bond definitions and hydrophobic fields^33^ are more parameterized due to the employment of field-based variant generated by Gaussian functions. Totally five Gaussian fields, including H-bond acceptor, electrostatic, steric, H-bond donor and hydrophobic were generated for all molecules and subsequently used to create QSAR models.

Compounds 1–37 were geometrically optimized by Ligprep module implemented in Schrodinger Suite 2013. After optimization, a single 3D structures of low energy was generated for all small moleculars and also many conformers/tautomers of a single ligand were generated via ionizing the ligands by EPIK module that create ionized states at pH range of 7 ± 2. Lonchocarpin was used as an alignment template for it had the best pIC_50_ value and the rest of the molecules were aligned to it by the method of flexible ligand alignment (see Supplementary Figure S3). Field-based QSAR tool of Schrodinger Suite 2013 was used to develop Gaussian-based QSAR models. Cytotoxic activity of 37 ligands from the Data set against the H292 cancer cell line was considered for building a QSAR model. Seventy-five percent of the data set molecules were randomly selected as training set. The obtained models were subjected to validation via prediction of test set ligands activity (compounds 5, 11, 15, 18, 23, 26, 30, 35, 36). Parameters such as performed using Gaussian based electrostatic, steric, hydrogen bond donor (HBD), hydrogen bond acceptor (HBA) and hydrophobic potential fields were calculated accordingly. For PLS regression analysis, pIC_50_ values of the molecules (see Supplementary Table S2) are considered as dependent variable and Guassian intensities are considered as independent variable. QSAR model was built and calculated by constructing with a 3D cubic lattice with 1 Å grid spacing, and can be extended by 3 Å beyond training set limits. Energies cutoff was set to ± 30 kcal/mol and the variable with standard deviation with <0.01 were eliminated.

### Molecular docking study

The three-dimensional crystal structure of navitoclax and Bcl-2 complex (PDB ID: 4LVT) along with other five Bcl-2 structures (PDB ID: 1YSW, 2O2F, 2O21, 4AQ3, 4IEH) was retrieved from the Protein Databank Bank (http://www.rcsb.org/). Before docking, the preparations of proteins and ligands (navitoclax, lonchocarpin and compound **12**, **15**, **23**, **26**, **33**) were performed following the standard protocol of the Protein Preparation and Ligprep Wizards, respectively, of the Schrodinger 2013 Suite. Docking studies were implemented via Grid-based Ligand Docking with Energetics (Glide) method^34^ in XP (Extra-Precision) mode that can give more precise G Score, by which the docking results were ultimately ranked. Many parameters, such as Coulombic, hydrogen bonds, van der Waals and polar interactions in the binding site, hydrophobic contacts, buried polar groups and energy penalties for freezing rotatable bonds, were taken into account to form the final G-Score. The Bcl-2 docking complexes of lonchocarpin and navitoclax were subject to mm-gbsa calculation to simulate binding energy, respectively.

### MM-GBSA calculation

The free energies of the Bcl-2-ligand complex of lonchocarpin and navitoclax were estimated with MM-GBSA method provided in Prime module of the Schrodinger 2013. Prime utilizes the VSGB 2.0 solvation model^35^ and the OPLS2005 force field^36^ to simulate these interactions.

### Animal experiment

The animal experiments were carried out according to the National Institute of Health Guide for the Care and Use of Laboratory Animals. And the use of experimental animals was approved by the Animal Ethics Committee of Shenyang Pharmaceutical University. Male Kunming mice, were housed in separate cages under standard conditions (20–24 °C), relative humidity (55% ± 5%) and a 12 h light/12 h dark cycle, and allowed to acclimatize for 3 days prior to the start of experiment. Cultured S180 cells were harvested and washed with sterilized PBS (pH 7.2) three times and resuspended at a density of 6 × 10^6^ cells/ml. Mice received subcutaneous implants of 0.2 mL/mouse on the right flank by means of hypodermic injection. Then the mice were divided randomly into 5 groups, including model, positive control and treatment groups with 10 mice in each group after inoculation for 24 h. Next, the treatment groups was given lonchocarpin at a dosage of 25, 50, and 100 mg/kg, while the positive control group was administrated with cyclophosphamide (CTX, 25 mg/kg) as a positive drug. Both lonchocarpin and CTX were dissolved in DMSO first and keeping the concentration of DMSO no more than 0.1% in physiological saline. Both groups received oral administrations via an intubation needle every day. Then the animals were observed after the drug administration everyday at the same time during the 12 days test period. The weight of the mice was recorded every day throughout the experiment. Finally, the mice were sacrificed by cervical dislocation just 2 h after the last administration. Then the tumor and spleen tissues were harvested and weighed immediately. After that the in *vivo* tumor inhibition ratio was calculated as: Inhibition ratio (%) = 100((A−B)/A), in which A represents the average tumor weight of the control group and B is recorded as the tumor weight of the administration group. Then, the spleen index was recorded using the following formula:

Spleen index = the final weight of spleen (mg)/the final average body weight (g) without tumor.

### Analysis of apoptosis by flow cytometry

Cell apoptosis was evaluated by Annexin V/PI staining assay and analyzed using an apoptosis detection kit (Keygen, Nanjing, China)^37^. Briefly, H292 cells were cultured at the density of 1 × 10^6^ cells/well. After 24 h, the cells were administrated with lonchocarpin (10 μM) and incubated for 12, 24, and 48 h. Both attached and floating cells were harvested at the arranged time point, and washed twice with ice-cold PBS, then resuspended in 100 μL binding buffer with Annexin V and PI for 15 min at 37 °C in darkness. Finally, the treated cells were analyzed by flow cytometry (Becton Dickinson, Franklin Lakes, NJ, USA) and Cell Quest software. The apoptosis rate was given by the following formula:

Apoptosis rate % = (number of apoptotic cells)/(number of total cells observed) × 100%.

### Western blotting

Western blotting was performed according to the method described in previous research^38^. Human lung cancer cells H292 were pretreated with DMSO (as a control) and lonchocarpin at 10 μM for 12 h, 24 h and 48 h. Then, the cells were harvested and then washed with PBS and lysed in a radio immune-precipitation assay buffer for 30 min. BCA protein assay kit (Byontime, Beijing, China) was used for determination of the total protein contents. And the protein samples were dissolved and seperated on 10% SDS-PAGE gels. After that the proteins were transferred to PVDF (polyvinylidene fluoride) (Bio-Rad, Hercules, CA) membranes and incubated sequentially with blocking buffer, primary antibodies, and horseradish peroxidase-conjugated secondary antibodies.

### Statistical analyses

The data were presented as mean ± SE (standard error) from three independent experiments. Statistical analysis was carried out using One-Way ANOVA (one-way analysis of variance) followed by Fisher’s least significant difference (LSD) test or Dunnet’s T3 test, using a statistical analysis software package (SPSS 19.0) and differences were considered statistically significant at P < 0.05.

## Electronic supplementary material

supplementary information
