# Supplementary material for: A natural chalcone induces apoptosis in lung cancer cells: 3D-QSAR, docking and an in vivo/vitro assay
Source: Sci Rep. 2017 Sep 6;7:10729. doi: 10.1038/s41598-017-11369-9 (PMC5587747; doi:10.1038/s41598-017-11369-9)
Supplement: Supplementary file 1 — supplementary information [file 41598_2017_11369_MOESM1_ESM.pdf]

A natural chalcone induces apoptosis in lung cancer cells: 3D-QSAR, docking and an *in vivo/vitro* assay

Gang Chen[1], Di Zhou[1], Xue-Zheng Li[2], Zhe Jiang[2], Chengyu Tan[3], Xiu-Yan Wei[4], Junhong Ling[5], Jing Jing[3], Fen Liu[1], Ning Li[1*]

[1] School of Traditional Chinese Materia Medica, Shenyang Pharmaceutical University, Shenyang 110016, China; State Key Laboratory for Chemistry and Molecular Engineering of Medicinal Resources, Guangxi Normal University, Guilin, China.

[2] Department of Pharmacy, Yanbian University Hospital, Yanji 133000, China

[3] College of Marine Technology and Environment, Dalian Ocean University, Dalian, China

[4] Department of Pharmacology, Shenyang Pharmaceutical University, Shenyang, 110016, China

[5] School of Pharmaceutical Engineering, Shenyang Pharmaceutical University, Shenyang, 110016, China

Cell line	H292	H522	H226	H358	A549	Calu-1	SW1573	H1792	H460	H1944
lonchocarpin	97.5	63.0	62.0	51.3	42.3	23.8	92.3	46.7	76.3	69.2
DDP	91.3	69.6	87.4	81.2	76.4	59.1	69.4	44.6	89.1	40.4

Table S1. The inhibiton rate (IR %) of lonchocarpin on 10 cell lines. Lonchocarpin: 100 μM; DDP: 10 μM

No	IC_{50} (μm)	pIC_{50}	Predicted Activity	Prediction Error	No	IC_{50} (μm)	pIC_{50}	Predicted Activity	Prediction Error
1	489.8	3.310	3.311	0.001	20	>1000	3.000	2.840	-0.160
2	316.2	3.500	3.061	-0.439	21	>1000	3.000	2.827	-0.173
3	>1000	2.988	3.074	0.086	22	>1000	3.000	2.843	-0.157
4	704.7	3.152	3.172	0.020	23	311.9	3.506	3.486	-0.020
5	772.7	3.112	3.253	0.141	24	393.5	3.405	3.249	-0.156
6	>1000	3.000	3.291	0.291	25	1000.0	3.000	3.232	0.232
7	613.8	3.212	3.182	-0.030	26	293.8	3.532	3.886	0.354
8	749.9	3.125	3.069	-0.056	27	>1000	3.000	3.143	0.143
9	974.9	3.011	3.099	0.088	28	630.9	3.200	3.054	-0.146
10	794.3	3.100	3.088	-0.012	29	1000.0	3.000	3.136	0.136
11	>1000	3.000	3.027	0.027	30	501.2	3.300	3.240	-0.061
12	100.0	4.000	4.050	0.050	31	>1000	3.000	3.094	0.093
13	615.2	3.211	3.281	0.070	32	776.2	3.110	3.281	0.171
14	629.5	3.201	3.218	0.017	33	215.8	3.666	3.281	-0.385
15	158.5	3.800	3.349	-0.451	34	10.0	5.000	4.804	-0.196
16	374.1	3.427	3.360	-0.067	35	61.5	4.211	4.794	0.583
17	944.1	3.025	3.159	0.134	36	50.0	4.301	3.967	-0.334
18	>1000	3.000	3.124	0.124	37	61.5	4.211	4.092	-0.119
19	>1000	3.000	3.254	0.254					

Table S2. IC_{50}, pIC_{50}, Predicted pIC_{50} and Prediction Error of 37 flavonoids.

PLS statistics Field Gaussian	Gaussian
SD	0.227
r^2	0.802
r^2cv	0.501
r^2 scramble	0.801
q^2	0.713
Field contributions (%)	
Steric	0.51
Electrostatic	0.05
Hydrophobic	0.23
HBA	0.14
HBD	0.07

Table S3 Summary of results obtained using Gaussian field based 3D-QSAR models. *SD* standard deviation of the regression, r^2 non-cross-validated correlation coefficient, r^2cv cross-validated correlation coefficient, q^2 LOO cross-validated correlation coefficient, *HBA* H-bond acceptor, *HBD* H-bond donor. A QSAR equation is generally acceptable if is r^2 approximately 0.9 or higher with the q^2 value that is higher than 0.5. An r^2cv is used as a diagnostic tool to evaluate the predictive power of an equation. SD is the sum of the squared deviations between the biological activities of each molecules and the mean activity of the training set of molecules.

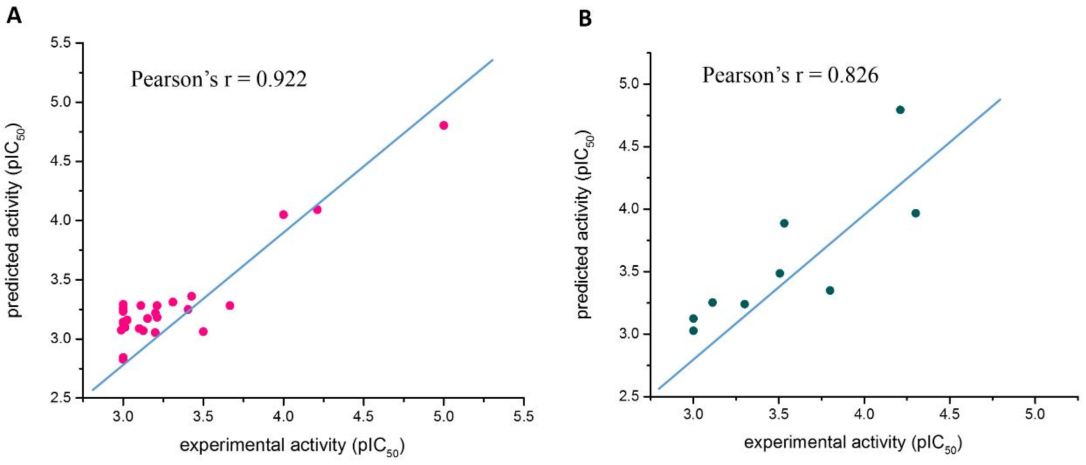

Figure S1. Relation between experimental and predicted activity values using field-based 3D-QSAR model. The activities were presented by pIC_{50} values. (A) training set molecules; (B) test set molecules.

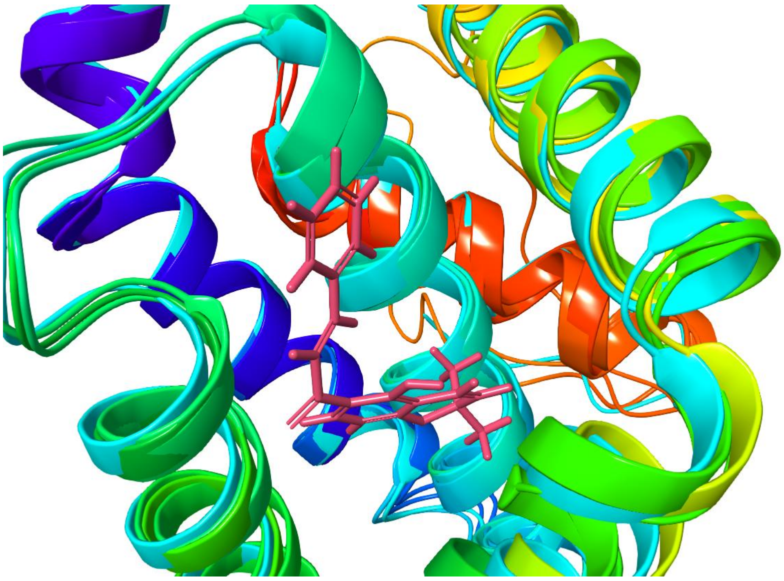

Figure S2. Stereo-view of six superimposed Bcl-2 protein structures. Six Bcl-2 protein structures (PDB: 1ysw, 2o2f, 2o21, 4aq3, 4ieh and 4lvt) were docked with lonchocarpin (dark pink). The docking results proved a similar way by which lonchocarpin inhibited the BH3-binding groove of the six Bcl-2 protein structures.

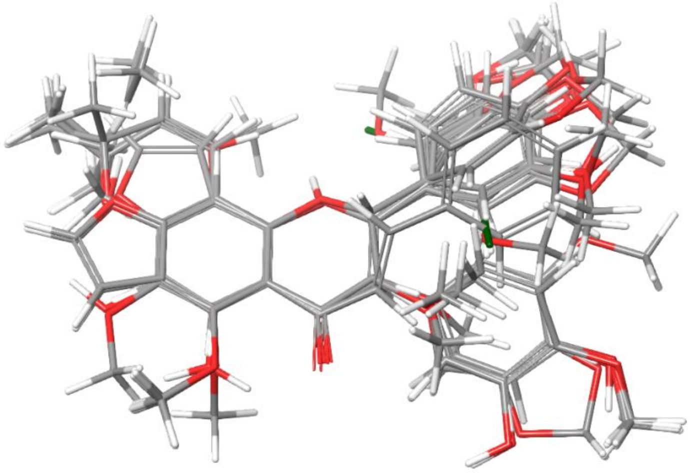

Figure S3. The training and test sets are aligned on lonchocarpin (34).

Extraction, isolation and identification of the compounds 1-37. The tested compounds were purified from dry stems of *P. pinnata* (L.) Pierre. Dry stems of *P. pinnata* (L.) Pierre (6.0 kg) were grounded and extracted with 95% ethanol (EtOH). And the EtOH extract was partitioned sequentially with dichloride methane (CH_2Cl_2), and normal butanol (n-BuOH). The CH_2Cl_2 extract (87 g) was subjected to repeated column chromatography over silica gel, RP-C18, Sephadex LH-20, preparative HPLC, to afford 37 known compounds. Among them, compounds **1**, **4**, **6**, **8**, **10**, **13**, **15**, **16**, **18-20**, **22**, **24-37** were reported in our previous work [1]. Compounds **2** (4′-hydroxypinnatin) [2], **3** (kanjone) [3], **5** (3′-hydroxyfuro[8,7:4″,5″] flavone) [4], **7** (2′-methoxyfurano (2″,3″:7,8)flavone) [5], **9** (6-hydroxy,3-methoxyfuro [8,7:4″,5″]flavone) [6], **11** (demethylate kanjone) [7], **12** (pongapinnol-C) [8], **14** (isopongaglabol) [9], **17** (pongaglabol) [10], **21** (genistein) [11], **23** (7,3′-dihydroxy-5′-methoxy isoflavone) [12] were isolated from stem of *P. pinnata* (L.) Pierre for the first time.

References

1. Li, J.Y. *et al*. Natural therapeutic agents for neurodegenerative diseases from a traditional herbal medicine Pongamia pinnata (L.) Pierre. *Bioorg Med Chem Lett.* **25**, 53-58 (2015).
2. Li, L. Y., Li, X., Shi, C., Deng, Z. W., & Lin, W. H. Studies on chemical constituents of semi-mangrove plant *Pongamia pinnata*. *Chinese Journal of Marine Drugs* **27**, 18-24 (2008).
3. Pathak, V. P., Saini, T. R., & Khanna, R. N. Isopongachromene, a chromenoflavone from pongamia glabra seeds. *Phytochemistry* **22**, 308-309 (1983).
4. Roy, D., & Khanna, R.N. Structure and synthesis of pongol a new component from seeds of *P. glabra*. *Indian Journal of Chemistry* **18B**, 525-528 (1979).
5. Pathak, V. P., Saini, T. R., & Khanna, R. N. A new furanoflavone from seeds of *Pongamia glabra*. *Planta Medica* **49**, 61 (1983).
6. Kamperdick, C., Dhuong, N.M., Sung, T.V., & Adam, G. Flavones and isoflavones from *Millettia ichthyostega*. *Phytochemistry* **44**, 577-579 (1998).
7. Ahmad, G., Yadav, P. P., & Maurya, R. Furanoflavonoid glycosides from *Pongamia pinnata* fruits. *Phytochemistry* **65**, 921-924 (2004).
8. Yadav, P. P., Ahmad, G., & Maurya, R. Furanoflavonoids from *Pongamia pinnata* fruits. *Phytochemistry* **65**, 439-443 (2004).
9. Talapatra, S. K., Mallik, A. K., & Talapatra, B. Isopongaglabol and 6-methoxyisopongaglabol, two new hydroxyfuranoflavones from *Pongamia glabra*. *Phytochemistry* **21**, 761-766 (1982).
10. Talapatra, S. K., Mallik, A. K., & Talapatra, B. Pongaglabol, a new hydroxyfuranoflavone, and aurantiamide acetate, a dipeptide from the flowers of *Pongamia glabra*. *Phytochemistry*, **19**, 1199-1202 (1980).
11. Marzouk, M. S., Ibrahim, M. T., El-Gindi, O. R., & Abou Bakr, M. S. Isoflavonoid glycosides and rotenoids from *Pongamia pinnata* leaves. *Zeitschrift fur naturforschung C* **63**, 1-7 (2008).
12. Ma, X. M., Wang, L. S., Guo, Y. J., & Guo, S. R. A new isoflavone from Huoxue Yiqi Tang. *China Journal of Chinese Materia Medica* **30**, 1159-1162 (2005).
